# Patients’ perspectives on ecologically sustainable health care in general practice: an experimental vignette and questionnaire study

**DOI:** 10.3399/BJGPO.2025.0041

**Published:** 2025-12-19

**Authors:** Eva H Visser, Evelyn A Brakema, Irene A Slootweg, Hedwig MM Vos, Marieke A Adriaanse

**Affiliations:** 1 Department of Public Health and Primary Care, Leiden University Medical Centre, Leiden, The Netherlands; 2 Department of Health, Medical and Neuropsychology of the Faculty of Social and Behavioural Sciences, Leiden University, Leiden, The Netherlands

**Keywords:** general practice, climate change, patient participation

## Abstract

**Background:**

Health care contributes substantially to climate change. GPs want to implement sustainable health care but are hesitant, worried that this may jeopardise their doctor–patient relationship. However, whether this concern is valid should be urgently assessed.

**Aim:**

To explore patients’ perspectives on sustainable health care in general practice.

**Design & setting:**

In 2022 and 2023, we performed an online study with Dutch adults using experimental vignettes and a questionnaire.

**Method:**

The vignettes described GP appointments for three health complaints with randomly allocated treatment advice, varying in sustainability and explanation, but with comparable health outcomes. The questionnaire assessed participants’ perspectives on sustainable health care in general practice. We analysed the vignettes using mixed-design analysis of variance (ANOVA) and the questionnaire using descriptive statistics and correlations.

**Results:**

In total, 801 participants completed the vignettes, and 397 the questionnaire. We found no difference on satisfaction with a doctor’s visit (*P* values >0.24) when comparing a sustainable and a less-sustainable treatment option. The effect of explicitly mentioning sustainability differed per health complaint (dyspnoea: no difference; knee pain: mean difference [MD] = 0.31, *P* = 0.002; erythema: MD = -0.23, *P* = 0.003). In the questionnaire, participants reported positive expectations, and trust in the GP and treatment when delivering sustainable health care, but were more neutral about the GP’s role.

**Conclusion:**

We found no indication that sustainable treatment advice leads to lower satisfaction with GP care. The effect of explicitly mentioning sustainability was minimal and differed per health complaint. When directly asked, participants were mainly positive about sustainable health care. These results could encourage GPs to introduce sustainable treatment advice, without worrying about negatively influencing patient satisfaction.

## How this fits in

GPs and other healthcare professionals increasingly want to implement sustainable health care, but are hesitant to do so, fearing that it will jeopardise their doctor–patient relationship. However, no studies have been conducted to assess how patients actually respond to sustainable health care in general practice. In this study among GP patients, we found the following: no indication that sustainable treatment advice, in scenarios with comparable health outcomes, leads to lower satisfaction with a doctor’s visit; that the effect of explicitly mentioning sustainability on satisfaction with a doctor’s visit had a minimal effect that differed per health complaint; and that participants were mainly positive about sustainable health care when reflecting on this topic in a questionnaire. These findings may encourage GPs to introduce sustainable treatment options in their consultations, without worrying about negatively influencing patient satisfaction.

## Introduction

Human activities place the planet’s ecosystems under severe pressure, resulting in climate change and other ecological crises.^1^ These crises affect our living environment and thereby increase human morbidity and mortality;^2^ the World Health Organization even considers climate change *‘the single biggest health threat facing humanity’*.^3^ Paradoxically, health care contributes substantially to these ecological crises through, for example, pollution and CO_2_ emissions.^4–8^ Therefore, healthcare professionals, including GPs worldwide,^9–11^ urgently call for a transition to ecologically sustainable health care; and it is also being incorporated into medical codes of conduct.^12,13^

Fortunately, there is a growing body of knowledge on how to mitigate the environmental footprint of health care. This includes, for example, knowledge on how to reduce surgery’s environmental harm,^14^ the carbon footprint of modes of delivery,^15^ or the environmental benefit of using dry powder inhalers compared with metered dose inhalers.^16^ The latter is even already included in GP guidelines from the Dutch College of General Practitioners.^17^

However, guidelines are frequently insufficient to change healthcare professional behaviour;^18^ knowledge does not automatically translate into motivation to change,^19,20^ let alone actual behaviour change, as these are dependent on other determinants as well.^21^ Healthcare professionals’ negative expectations about patients’ responses has been identified as a key barrier to providing sustainable health care ,^22–24^ as well as corresponding concerns about jeopardising their doctor–patient relationship. However, exploration of patients’ responses towards sustainable health care has been limited and, to our knowledge, has not yet occurred in the context of general practice,^25–28^ so it is unclear whether or not these concerns are warranted. Therefore, we aimed to study patients’ responses to sustainable health care in general practice and the potential influence on satisfaction with care.

## Method

We conducted an online study including experimental vignettes and a questionnaire. The study was approved by the Departmental Ethical Review Board of Leiden University Medical Center (#22-3046) and preregistered on AsPredicted (https://aspredicted.org/2df6w.pdf) Data and syntaxes are available on the Open Science Framework (https://osf.io/pvd4u/).

### Study design

The study consisted of two parts, starting with experimental vignettes with random allocation, without disclosure of the topic of sustainable health care; and, after disclosure, a questionnaire, similar for all participants.

We chose short written experimental vignettes because real-life observations were not feasible (owing to limited sustainable GP treatment options), to ensure accessibility for most participants (vignettes are in B1 level Dutch; an independent user level of proficiency),^29^ and for their ability to control variables^30^ and avoid potential bias as present in questionnaires (for example, social desirability bias).^31,32^

We collected data in two independent waves; wave one in October 2022 and wave two in March 2023. We report data for the experimental vignette study of both waves and the questionnaire data of wave two. The questionnaire data from wave one were excluded owing to a coding error, where the 5-point Likert scale was visually reversed (descending instead of ascending order).

See Supplementary Figure S1 for a study flow diagram.

### Participants

We included a representative sample of Dutch adults, based on age, gender, geographical distribution, and educational level. As all Dutch citizens are patients at a GP surgery, the participants in this sample are all GP patients; although not attending an appointment at the time of the survey. Recruitment and administration was performed by a research agency (Flycatcher Internet Research). We chose Flycatcher because of its experience and the possibility of easily obtaining a representative sample. Further details on Flycatcher are available in the Supplementary Data. We obtained digital informed consent before participation.

### Materials

#### Baseline demographics

Baseline demographics included age, gender, education level, type of living area, having (grand)children, trust in the GP, number of GP visits, and self-rated health status.

#### Experimental vignettes

The vignettes were developed in collaboration with experts on sustainable health care, GPs, and GP trainees. We did not inform participants about the study’s purpose and the random allocation, before they saw the vignettes. The experimental vignettes presented each participant with three scenarios about a GP visit. Each scenario described a different health complaint (that is, ‘Health complaint’): dyspnoea, knee pain, and erythema. These health complaints have at least two treatment options that have comparable health outcomes, but, with current knowledge, differ in their environmental impact.^17,33,34^ Participants were randomly assigned to one of four predefined possible doctor’s advice (that is, ‘Type of advice’) that were then applied to all three scenarios. This advice varied in whether it was more sustainable or less sustainable, and whether sustainability was named as an argument:

– the less-sustainable advice: only the less-sustainable treatment option was offered;
– the sustainable advice: only the more-sustainable treatment option was offered;
– the sustainable with alternative advice: the more-sustainable treatment option was offered, while making explicit that there is another option;
– the sustainable made explicit advice: the more-sustainable treatment option was offered, while making explicit that there is another option, with sustainability as an explicit argument for suggesting this advice.

See Table 1 for examples.

**Table 1.** Example of all type of advice options from the dyspnoea vignette

Less-sustainable advice
You visit your GP because you sometimes feel short of breath. After taking your medical history and performing a physical examination, the GP diagnoses you with a lung disease such as asthma or COPD. The doctor wants to start you on medication. They recommend a metered dose inhaler or puffer to help you feel less short of breath.
**Sustainable advice**
You visit your GP because you sometimes feel short of breath. After taking your medical history and performing a physical examination, the GP diagnoses you with a lung disease such as asthma or COPD. The doctor wants to start you on medication. They recommend a dry powder inhaler to help you feel less short of breath.
**Sustainable with alternative advice**
You visit your GP because you sometimes feel short of breath. After taking your medical history and performing a physical examination, the GP diagnoses you with a lung disease such as asthma or COPD. The doctor wants to start you on medication. There are different types of medication available: a metered dose inhaler or puffer or a dry powder inhaler They recommend a dry powder inhaler to help you feel less short of breath.
**Sustainable made explicit advice**
You visit your GP because you sometimes feel short of breath. After taking your medical history and performing a physical examination, the GP diagnoses you with a lung disease such as asthma or COPD. The doctor wants to start you on medication. There are different types of medication available: a metered dose inhaler or puffer or a dry powder inhaler They recommend a dry powder inhaler to help you feel less short of breath. The GP tells you that this type of medication is better for the environment than the metered dose inhaler.

COPD = chronic obstructive pulmonary disease

This resulted in a 3×4 design: the three different health complaints assessed differences within subjects; the four types of advice assessed differences between subjects.

After each vignette we asked participants to rate their ‘Satisfaction with the doctor’s visit’ averaged from four items assessing the acceptability of the treatment, trust in the GP, confidence in the treatment, and the feeling that their health is a priority to the GP (ascending 5-point Likert scale).

After completing the vignettes, we asked participants to indicate whether they had experienced similar health complaints and treatment advice.

#### Sustainable Healthcare Questionnaire in General Practice (SHQ_GP)

We developed the Sustainable Healthcare Questionnaire in General Practice (SHQ_GP) to assess patients’ general perspective on sustainable health care within the context of general practice. The questionnaire included 15 items, assessing seven constructs based on broader literature on patient satisfaction and healthcare professional behaviour (ascending 5-point Likert scale).

#### Overall opinion on climate change

We administered five items of the ‘single items scale’ of van Valkengoed *et al* to assess the overall opinion on climate change (ascending 5-point Likert scale).^35^

The experimental vignettes and SHQ-GP (Dutch and English), and development rationale are available in the Supplementary Data.

### Statistical analysis

We used IBM SPSS Statistics (version 29) for statistical analyses. We present the demographic variables using frequencies, percentages, medians, and ranges.

#### Vignettes

We conducted χ^2^ and Kruskall–Wallis tests to check for successful randomisation. We assessed the effect of ‘Type of advice’ (between subjects) and ‘Health complaint’ (within subjects) on ‘Satisfaction with the doctor’s visit’ using mixed-design ANOVA, presenting the Huynh-Feldt estimates. We used Spearman’s rho to explore the relationship between demographics, experience with the diseases, overall opinion on climate change, and ‘Satisfaction with the doctor’s visit’ for each ‘Health complaint’.

#### Questionnaire

We analysed the SHQ_GP using percentages, medians, interquartile ranges (IQR); and Cronbach’s alpha per construct. We used Spearman’s rho to explore the relationship between demographics, overall opinions on climate change, and the constructs.

Adjustments to our preregistration and rationale for methodological choices are available in the Supplementary Data.

## Results

We sent the survey to 1330 participants, of whom 801 (wave one and two) completed the experimental vignettes and 397 (wave two) completed the SHQ_GP.

### Demographics

Participants’ average age was 53 years and gender was equally distributed (50.7%, *n* = 406 males). Participants indicated a mean of two GP visits per year. Demographics are available in Table 2.

**Table 2.** Demographic variables

	All participants (*n* = 801)									
Median age, years [range]	53 [18–95]									
Gender	Male		Female			Non-binary or genderqueer			Other	
	50.7% (*n* = 406)		49.1% (*n* = 393)			0.2% (*n* = 2)			0.0% (*n* = 0)	
Education level	Lower			Medium				High		
	26.5% (*n* = 212)			41.3% (*n* = 331)				32.2% (*n* = 258)		
Type of living area	Urban					Rural				
	52.8% (*n* = 423)					47.2% (*n* = 378)				
Having children, yes	61.8% (*n* = 495)									
Having grandchildren, yes	30.3% (*n* = 243)									
I trust my GP	Strongly disagree	Disagree			Neither agree nor disagree		Agree			Strongly agree
	6.6% (*n* = 53)	6.0% (*n* = 48)			11.4% (*n* = 91)		47.2% (*n* = 378)			28.8% (*n* = 231)
Median number of GP visits per year [range]	2 [0–50]									
Self-rated health status	Poor	Fair			Good		Very good			Excellent
	2.6% (*n* = 21)	21.5% (*n* = 172)			52.3% (*n* = 419)		19.6% (*n* = 157)			4.0% (*n* = 32)

### Experimental vignettes

#### Randomisation check

Participants were equally distributed across the types of advice (*n* = 198 to *n* = 204). We found no statistically significant differences between the four groups (Type of advice) on demographics, control variables, and overall opinion on climate change, except on ‘Number of GP visits per year’ (*P* = 0.04). Conforming to the preregistered research plan (AsPredicted, https://aspredicted.org/2df6w.pdf ), we included this variable as covariate in the main analysis, but report the analyses without this covariate as it was not statistically significant.

#### Main analysis

The mixed-design ANOVA on ‘Satisfaction with the doctor’s visit’ did not yield a statistically significant main effect of ‘Type of advice’ (*P* = 0.41). The within subjects’ effect of ‘Health complaint’ was statistically significant (*F* [1.91, 1521.52] = 326.98, *P*<0.001, partial η^2^ = 0.29), with the highest scores for erythema (*M* = 3.87, standard deviation [SD] = 0.77), then for dyspnoea (*M* = 3.78, SD = 0.87), and the lowest score for knee pain (*M* = 3.12, SD = 0.99). The ‘Type of advice’ x ‘Health complaint’ interaction was also statistically significant (*F* [5.73, 1521.52] = 8.89, *P*<0.001, partial η^2^ = 0.03). Pairwise comparisons were conducted to decompose the interaction.

**Figure 1.**
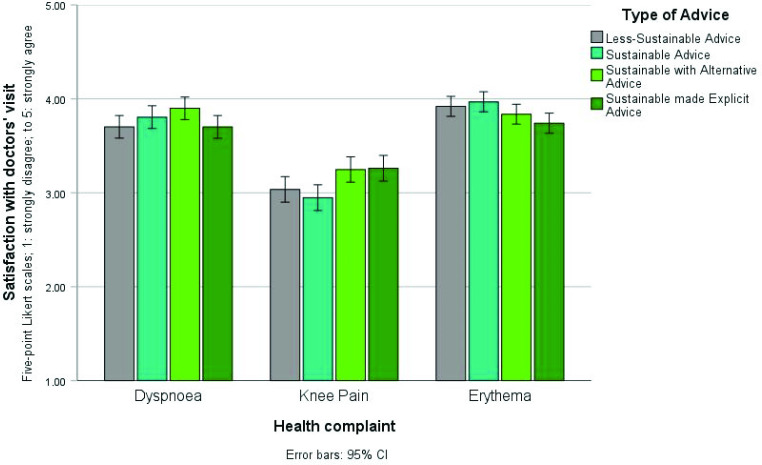
‘Satisfaction with the doctor’s visit’ (ascending 5-point Likert scale) per ‘health complaint’, with separate bars per ‘type of advice’

These exploratory pairwise comparisons within each of the ‘Health complaints’ revealed that within dyspnoea, the sustainable with alternative advice (*M* = 3.90, standard deviation [SD] = 0.85) received statistically significant higher scores on satisfaction than the less-sustainable advice (*M* = 3.70, SD = 0.88; mean difference [*MD]* = 0.20, *p* = 0.02), as well as sustainable made explicit advice (*M* = 3.70, SD = 0.99; *MD* = 0.20, *p* = 0.02). All other *P values* were >0.24. Within knee pain, the sustainable with alternative advice (*M* = 3.25, SD = 0.98) scored statistically significant higher on satisfaction as compared with both the less-sustainable advice (*M* = 3.04, *SD* = 1.01; *MD* = 0.21, *p* = 0.03) as well as the sustainable advice (*M* = 2.95, SD = 0.96; *MD* = 0.30, *p* = 0.002). Furthermore also the sustainable made explicit advice (*M* = 3.26, SD = 0.98) scored statistically significant higher on satisfaction as compared with both the less-sustainable advice (*M* = 3.04, *SD* = 1.01; MD = 0.02, p = 0.02) as well as the sustainable advice (*M* = 2.95, SD = 0.96; MD = 0.31, p = 0.002). All other *P-values* were>0.37. Lastly, within erythema, the sustainable made explicit advice (*M* = 3.74, SD = 0.89) scored statistically significant lower than both the less-sustainable advice (*M* = 3.92, SD = 0.76; MD = -0.18, p = 0.02) and the sustainable advice (*M* = 3.97, SD = 0.64; MD = -0.23, p = 0.003). All other *P-*values were>0.09. All pairwise comparisons are available in Supplementary Table S1 and Figure 1 gives a visual representation with mean scores on ‘Satisfaction with a doctor’s visit’ across the ‘Health complaints’ and ‘Types of advice’.

Correlations between demographics, overall opinion on climate change, and health complaints, are shown in Supplementary Table S2. There are no statistically significant correlations with prior experience with a disease and satisfaction with the doctor’s visit in the corresponding vignette.

#### Sustainable Healthcare Questionnaire in General Practice (SHQ_GP)

**Figure 2.**
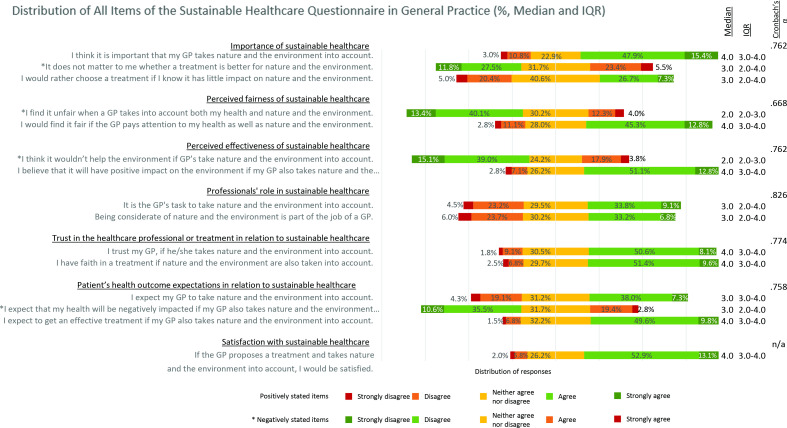
Distribution of scores of all SHQ_GP items clustered per construct. ^a^Negatively stated items

The SHQ-GP shows that participants are generally neutral to positive about sustainable health care, with low percentages expressing negative opinions to the statements (strongly disagree, or strongly agree in negatively stated items <6.0%). Overall, participants reported positive expectations, trust in the GP and treatment when delivering sustainable health care, but are more neutral about the role or task of the GP in sustainable health care. A complete overview can be found in Figure 2, showing the percentages per given answer, with the median and IQR per item, and Cronbach’s alpha per construct.

Correlations between demographics and SHQ_GP constructs in Table 3 show that age correlated positively with the perceived role of the GP in sustainable health care. For gender, there is a small correlation indicating that women or non-binary or genderqueer people have a more positive stance regarding importance and effectiveness of sustainable health care. Finally, an increase in education level is associated with a more positive stance regarding fairness and effectiveness. These correlations showed no consistency over the constructs. There is no statistically significant correlation between demographic variables and trust, expectations, or satisfaction with treatment. See Supplementary Table S3 for the complete correlation table.

**Table 3.** Correlation table of demographic variables (age, gender, education level) with SHQ_GP constructs

		Demographics			Constructs in SHQ_GP						
		Age in years	Gender	Education level	Importance of sustainable health care	Perceived fairness	Perceived effectiveness	Health professional role	Trust	Expectation	Satisfaction with treatment
**Demographics**											
Age in years	r_s_	--									
Gender	r_s_	-0.289^a^	--								
Education level in three categories	r_s_	-0.323^a^	0.053	--							
**Constructs in SHQ_GP**											
Importance of sustainable health care	r_s_	0.066	0.133^a^	0.069	--						
Perceived fairness	r_s_	-0.061	0.061	0.155^a^	.0.684^a^	--					
Perceived effectiveness	r_s_	-0.09	0.118^b^	0.194^a^	0.714^a^	0.719^a^	--				
Health professional role	r_s_	0.197^a^	-0.03	0.004	0.714^a^	0.545^a^	0.535^a^	--			
Trust	r_s_	-0.003	-0.037	0.076	0.626^a^	0.671^a^	0.616^a^	0.548^a^	--		
Expectation	r_s_	0.095	0.009	0.051	0.766^a^	0.740^a^	0.732^a^	0.722^a^	0.742^a^	--	
Satisfaction treatment	r_s_	-0.073	0.045	0.071	0.595^a^	0.646^a^	0.621^a^	0.473^a^	0.687^a^	0.643^a^	--

^a^Correlation is statistically significant at the 0.01 level (2-tailed). ^b^Correlation is statistically significant at the 0.05 level (2-tailed). SHQ_GP = Sustainable Healthcare Questionnaire in General Practice

## Discussion

### Summary

In this study we explored patients' perspectives on sustainable health care in general practice and the potential influence of giving sustainable treatment advice on satisfaction with the doctor’s visit, in scenarios with comparable health outcomes. We found no differences in satisfaction when a more-sustainable treatment option is advised compared with a less-sustainable treatment option. The effect of explicitly naming sustainability as an argument for choosing a treatment option, compared with advising the sustainable option without such an explanation, was small and varied per health complaint. Lastly, most participants responded relatively positively when asked about sustainable health care in general practice.

### Strengths and limitations

We conducted this study combining expertise from general practice, sustainable health care, and behavioural science. This allowed us to combine the reality of everyday medical practice, ambitions regarding sustainable health care, and aligning these with behavioural science knowledge and methodology (for example, experimental vignettes). In addition, the large and representative sample strengthens our confidence regarding the robustness of our findings. Lastly, the use of experimental vignettes and no disclosure about the topic (that is, sustainable health care) provides the currently closest approximation to the actual situation.

We also recognise there are limitations to our study. First, our findings rely on participants’ hypothetical perspectives as obtaining real-life behavioural responses was not feasible. This may have reduced the realism of our study, and, consequently, its external validity.^32^ However, our primary interest was in the relative differences between types of advice, rather than their absolute value. Second, patients were not involved in the vignette design, which may have led to less alignment with participants' perspectives. Future studies should preferably involve all stakeholders, including patients, in their design. Third, it may be possible that participants in the sustainable made explicit advice group inferred the study aim based on the design of the vignettes, which could have potentially influenced the data. Future studies should consider asking if the aim of the study is clear after the vignettes to assess this. Fourth, despite our attempt to use a representative sample, the sample exhibited a lower frequency of doctor visits than the median reported in national databases.^36^ This discrepancy may indicate that our sample is healthier than the average population or may be the result of recall bias. Still, these findings must be interpreted with caution in patients who visit their GP more frequently. Lastly, we included non-acute vignettes with similar health outcomes. Future research should replicate our findings with different patient outcomes and in other care settings.

### Comparison with existing literature

#### Satisfaction with a doctor’s visit is not negatively — nor positively — influenced by recommending a sustainable treatment option

Regardless of whether participants deliberately reflected on sustainable health care in general practice (questionnaire) or when comparing patient responses who were randomly assigned to less-sustainable versus sustainable treatment options with comparable health outcomes in hypothetical GP scenarios, without making the topic of sustainable health care explicit (experimental vignettes), sustainable health care did not yield negative responses regarding patients’ satisfaction. The concerns about undermining trust, as expressed by Resnik and Pugh,^37^ or the concerns of jeopardising the doctor–patient relationship,^24^ thus do not seem grounded in patient opinion. In fact, our results can be seen as fertile ground to further encourage and support the implementation of sustainable health care.

#### Explicitly naming sustainable health care as an argument to support the decision to prescribe a sustainable treatment option has small, mixed effects across the scenarios

When explicitly naming sustainability as an argument compared with not naming it explicitly, patient satisfaction differed per health complaint; it was marginally higher for knee pain, indifferent in dyspnoea, and lower in erythema. This difference may stem from patients' varying perspectives on the complaints and treatment options presented. For example, the knee pain vignette emphasised symptom relief, while erythema involved causal treatment and dyspnoea concerned potential life-saving medication. Recognisability could also explain the difference, with knee pain being most familiar and treatable with over-the-counter medication, dyspnoea more commonly encountered, and erythema less common. However, exactly why this difference exists across health complaints is yet to be determined in future studies.

Present findings leave the ethical question of how much we, as GPs, are willing to trade off in terms of patient satisfaction when this leads to an increase in awareness regarding sustainable health care, also for the sake of health. Especially since a 2015 study found that primary care physicians are considered the most trusted source when it comes to health information related to global warming, making GPs potentially important stakeholders in raising awareness.^38^

#### Patients respond neutral to positively to sustainable health care in general

The SHQ_GP showed an overall neutral to positive response from participants towards sustainable health care in the context of general practice. This seems to be largely independent of presumed indicators of a higher commitment to ecological crises, such as lower age, female sex, or higher education level.^39^ Most participants expected to be satisfied with receiving sustainable health care, reported positive expectations towards sustainable health care, and trust in both the GP and treatment when sustainable health care was delivered. Most participants also indicated to perceive sustainable health care as important. Notably, there is less consensus regarding the role or task of the GP in the context of sustainable health care; participants appear to exhibit a more neutral stance on this matter. Despite the majority having a neutral to positive attitude toward sustainable health care, there is also a non-negligible group of people (outspokenly) negative about the importance and expectations regarding sustainable health care. This makes it even more important to have situational awareness and to match the treatment advice, and in this case in particular the explanation, to the individual patient.

### Implications for research and practice

The patients’ perspective on sustainable health care in general practice tends to be generally positive. This suggests that we, as GPs, can start changing our behaviour and advise sustainable treatment options with comparable health outcomes, without worrying about negatively influencing patient satisfaction. Explicitly discussing sustainability as an argument should be treated with more care. Future research will have to focus on whether our findings can be extrapolated to other settings or to treatments with non-comparable health outcomes; or replicate our study with real-world observations.

## Data

The study was preregistered on AsPredicted (https://aspredicted.org/2df6w.pdf). Data and syntaxes will be made available on the Open Science Framework (https://osf.io/pvd4u/) after publication.
